# Application and Challenge of 3rd Generation Sequencing for Clinical Bacterial Studies

**DOI:** 10.3390/ijms23031395

**Published:** 2022-01-26

**Authors:** Mariem Ben Khedher, Kais Ghedira, Jean-Marc Rolain, Raymond Ruimy, Olivier Croce

**Affiliations:** 1 Bacteriology Laboratory, Archet 2 Hospital, CHU Nice, 06000 Nice, France; 2 Institute for Research on Cancer and Aging Nice (IRCAN), CNRS, INSERM, Université Côte d’Azur, 06108 Nice, France; 3 Laboratory of Bioinformatics, Biomathematics and Biostatistics, Institute Pasteur of Tunis, Tunis 1002, Tunisia; kais.ghedira@pasteur.tn; 4 IRD, APHM, MEPHI, IHU-Méditerranée Infection, Aix Marseille Université, 13005 Marseille, France; jean-marc.rolain@univ-amu.fr; 5 Centre Méditerranéen de Médecine Moléculaire (C3M), INSERM, Université Côte D’Azur, 06108 Nice, France

**Keywords:** long-read sequencing, whole-genome sequencing (WGS), bacterial genomes, next-generation sequencing, genomics, metagenomics, metatranscriptomics, transcriptomics

## Abstract

Over the past 25 years, the powerful combination of genome sequencing and bioinformatics analysis has played a crucial role in interpreting information encoded in bacterial genomes. High-throughput sequencing technologies have paved the way towards understanding an increasingly wide range of biological questions. This revolution has enabled advances in areas ranging from genome composition to how proteins interact with nucleic acids. This has created unprecedented opportunities through the integration of genomic data into clinics for the diagnosis of genetic traits associated with disease. Since then, these technologies have continued to evolve, and recently, long-read sequencing has overcome previous limitations in terms of accuracy, thus expanding its applications in genomics, transcriptomics and metagenomics. In this review, we describe a brief history of the bacterial genome sequencing revolution and its application in public health and molecular epidemiology. We present a chronology that encompasses the various technological developments: whole-genome shotgun sequencing, high-throughput sequencing, long-read sequencing. We mainly discuss the application of next-generation sequencing to decipher bacterial genomes. Secondly, we highlight how long-read sequencing technologies go beyond the limitations of traditional short-read sequencing. We intend to provide a description of the guiding principles of the 3rd generation sequencing applications and ongoing improvements in the field of microbial medical research.

## 1. Transformation of Genome Sequencing Landscape

### 1.1. Emergence of Nucleic Acid Sequencing

The knowledge of the DNA sequences of an organism is one of the cornerstones of modern biological science. Indeed, the sequence determination of various species has facilitated the study of genome content, genes, their encoding products and the relationship between them.

The first molecules to be sequenced were ribonucleic acids (RNAs) because of their simpler nature and smaller size. The very first RNA sequenced—the yeast alanine transfer RNA—dates back to 1965 [1] and was followed in parallel by Frederick Sanger’s development of a technique using radioactively labeled partial digest fragments separated in two dimensions by migration on various membranes under high voltage. However, the first revolution in sequencing occurred in 1977, thanks once again to Frederick Sanger [2]. He introduced the use of dideoxyribonucleotides (ddNTPs), analogues of the nucleotides that make up DNA. Improvements in this technique led to the first automated sequencers: fluorophores replaced radioactivity, and capillary electrophoresis separation replaced gels, while Roger Staden showed the performance of computer programs used to assemble sequences [3]. This first generation of sequencers was able to generate sequences of about 1000 base pairs at maximum.

### 1.2. Awake of Microbial Genomics

The revolution in bacterial genome sequencing occurred in 1995 when Craig Venter, Hamilton Smith and their associates performed the first sequencing of the whole genome of a non-pathogenic *Haemophilus influenzae* strain [4] using the Sanger method. Sanger sequencing was an accurate technique, but it is labor-intensive, time-consuming, relatively expensive and has a low throughput, thereby limiting its applications for whole-genome sequencing [5]. Indeed, this technology has difficulties obtaining certain gene sequences and even more difficulty obtaining complete genomes.

**Figure 1:**
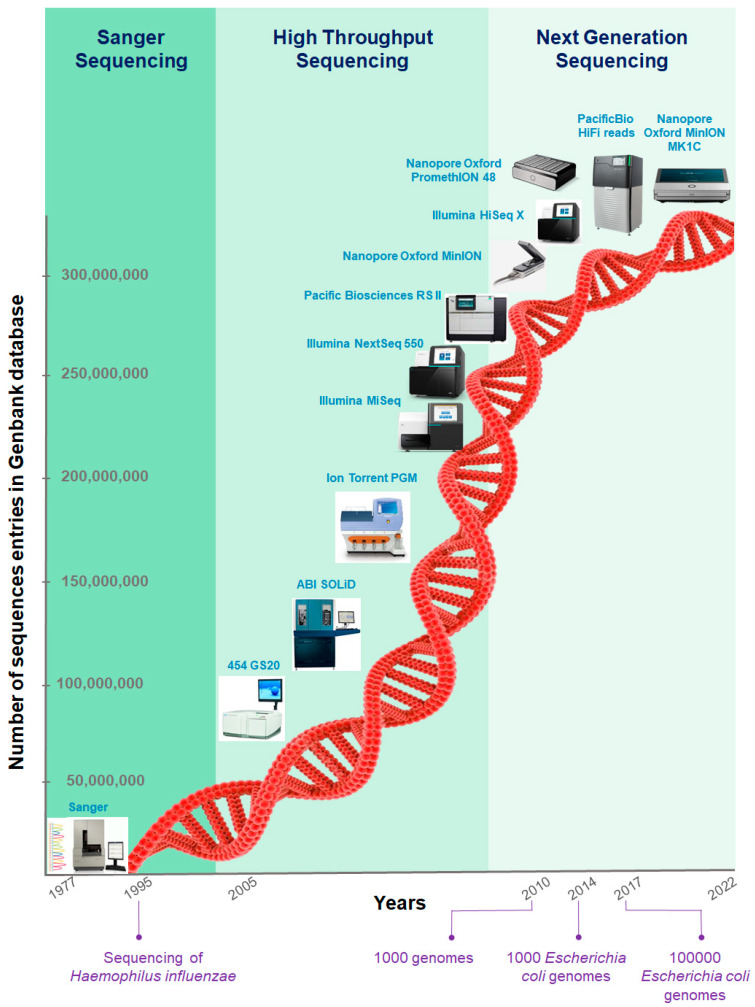
Overview of the evolution of bacterial genome sequencing.

The limit of Sanger’s sequencing in terms of low throughput and complexity has been overcome after the release of the human genome project by the development of high throughput sequencing technologies (HST) [6]. Several reviews have widely addressed the HTS strategies [7,8,9]. The depth of sequencing has made great leaps compared to Sanger technology, even if the maximum read length dropped below a few hundred base pairs. The evolution of HST provided next-generation sequencing (NGS) and reached the bacteriology domain [9,10]. Indeed the emergence of these NGS platforms started with the Life Sciences company that initiated a new turn in sequencing technologies with the launch of its high throughput sequencer “454 GS Flex” [11]. Many laboratories have been able to access this technology, either directly by buying sequencers or by using the service of companies or other partner laboratories that have acquired such machines. For example, the 454 sequencer from Life Sciences (Darmstadt, Germany) released in 2005 had a reagent cost of around $10 per megabase, a cost that rapidly decreased in the following years. Later on, sequencers such as the 454 from Roche (Basel, Switzerland) or the Solid from Life Technologies (Darmstadt, Germany) gave way to sequencers such as the Ion Torrent from Thermo Fisher (Waltham, MA, USA) [12] or the various models of sequencers from Illumina company (San Diego, CA, USA) (MiSeq, NextSeq and HiSeq) [13], which further reduced costs while increasing the quality of the data produced [12]. These new sequencers are still producing short reads (2 × 300 bp maximum for Illumina and 600 bp for IonTorrent) (Illumina, San Diego, CA, USA) but are based on distinct approaches and technologies such as the use of a bridge polymerase chain reaction (PCR) amplification and the detection of fluorescent light released after the incorporation of labeled nucleotides [12]. Thanks to these technologies, the total cost to sequence a complete bacterial genome has become affordable to many more people, which has contributed to opening the doors of genomics. A chronology that encompasses the various sequencing revolutions is highlighted in Figure 1.

Such sequencers and their evolution drastically accelerated the numbers of completely sequenced bacterial genomes [14]. For instance, thousand genome sequencing was achieved in 2007, the thousandth genome of *Escherichia coli* was achieved in 2014 and the 100th thousandth genome in 2017. Currently, more than 376,000 bacterial genomes projects are deposited and available in public databases (https://www.ncbi.nlm.nih.gov/genome/browse/#!/prokaryotes/, accessed on 25 January 2021).

### 1.3. Short-Read Sequencing Limitations

The short-read DNA sequencing process is mainly based on the clonal amplification of adaptor-ligated DNA fragments on the surface of a glass flow cell [12]. A cyclic reversible termination strategy is used for base reading, sequencing the template strand one nucleotide at a time through a progressive cycle of base incorporation, which is followed by an imaging step to identify the incorporated nucleotide at each cluster and by a cleavage step. To determine the added nucleotide by fluorescent imaging and the removal of unincorporated bases, short-read DNA sequencing platforms use fluorescently-labeled 3′-O-azidomethyl-dNTPs to pause the polymerization reaction [15]. The fluorescent moiety and the 3′ block are removed after scanning the flow cell with a coupled-charge device (CCD) camera, and the process is repeated. Currently, Illumina (San Diego, CA, USA) dominate the NGS market with several device models.

Regarding de novo genome assemblies, the evolution of bioinformatics tools in association with the increase of the sequencing depth partially compensate for the limitations due to the length of short reads. However, the multiple copies of some genes or repeated elements such as the rRNA operon in bacteria [16] cannot be easily resolved. This leads to incomplete assemblies with a draft quality, composed of fragmented sequences (contigs or scaffolds) or unresolved sequences (gaps) [17]. Even though the total genes content is mostly sequenced in a fragmented genome, the contiguous structure remains unknown, and the repeated areas are still poorly defined or badly located. This has the consequence of limiting some analyses such as the detection of horizontal gene transfers, the studies of multiple operons, the discovery of particular gene clusters or the accurate identification of mobile elements in a given organism. Moreover, ambiguous assemblies due to short reads can generate errors that could compromise the prediction of protein-coding sequences (CDSs) or genes annotation [18]. Sequencing that uses paired-end or especially mate-paired techniques (fragments usually between 1 kb to 3 kb lengths) partially compensate for these weaknesses. However, it still fails when some repetitive sequences are longer than the maximum fragment size sequenced (i.e., copies of bacterial rRNA operons exceeding 5 kb).

### 1.4. Long-Read Sequencing Developments

New sequencer machines appeared in 2011, proposing single-molecule sequencing technologies able to sequence over 10 kb of length. These long-read sequencings offer great advantages, including the ability to resolve repeats sequences [19].

Two technologies currently dominate the long-read sequencing space: ‘Pacific Biosciences’ (PacBio (Pacific Biosciences, Menlo Park, CA, USA)) single-molecule real-time (SMRT) sequences [20] and ‘Oxford Nanopore Technologies’ (ONT (Oxford Nanopore Technologies, Oxford, UK)) nanopore sequencing (Company history n.d.) which were commercially released in 2011 and 2014, respectively. The SMRT PacBio (Pacific Biosciences, Menlo Park, CA, USA) was the first long-read sequencer to be widely used. It is able to detect a single DNA molecule in real-time [21]. SMRT is based on DNA replication, utilizing the detection of released fluorophores as each nucleotide is added in the sequencing process. PacBio’s SMRT (Pacific Biosciences, Menlo Park, CA, USA) sequencing enables the real-time detection of nucleotide incorporation events during the elongation of the replicated strand from the non-amplified single-stranded template. The Nanopore from ONT (Oxford Nanopore Technologies, Oxford, UK) appeared later in 2014, and the MinIon (Oxford Nanopore Technologies, Oxford, UK) model was the first portable sequencer with a weight of only 100 g. The principle is based on a membrane including nanopores (transmembrane proteins), to which a low voltage is applied. The membrane detects the translocation signals, i.e., it acts as a nucleic acid counter by detecting the interruption to the current as they pass through the pore. Nanopore is less expensive than PacBio (Pacific Biosciences, Menlo Park, CA, USA). On the other hand, PacBio (Pacific Biosciences, Menlo Park, CA, USA) retains the advantage of better sequencing quality.

This third-generation sequencing has opened exciting avenues in genomics and has become suitable for an increasing number of applications. These capabilities have significantly improved accuracy and yield advances, making long-read sequencing key to a wide range of genomics applications for model and non-model organisms [22]. The advent of long-read technologies has the potential to transform clinical research and genomics analysis applications. An overview of the main advantages of long-read sequencing compared to short-read sequencing approaches are listed in Table 1.

These technologies enhance de novo genome assembly allowing us to obtain contiguous bacterial genomes with good reliability, an accurate reconstruction of gene order and orientation, without conducting complex finishing steps [23]. Loman et al. showed the feasibility of assembling a complete bacterial genome (*Escherichia coli* K-12 MG1655) in good quality using only long-reads produced by a MinION sequencer (Oxford Nanopore Technologies, Oxford, UK) [23] since long-read technology is now mainly used to obtain complete genomes.

Long-read technology also has other advantages. It improves the identification of transcription isoforms [24], the detection of structural variants [25], enables the direct detection of haplotypes and even whole chromosome phasing [26,27]. Finally, it makes it possible to sequence single molecules in real-time, avoiding DNA amplification which could be a bias inherent to second generation sequencing [28]. The ease of use of the Nanopore MinIon (Oxford Nanopore Technologies, Oxford, UK) has allowed sequencing to be performed with limited resource environments and in situ natural environments [29]. The machine also presents the opportunity to decentralize sequencing with fast run times, accurate performance and the ability to simply drop a sample onto the sequencer without any preparation. The consequences of this evolution towards long-read sequencing has given rise to numerous studies [30,31,32,33].

The affordability and usability of long-read single-molecule sequencing instruments has facilitated new real-time applications of disease outbreaks [34]. As shown by Joshua Quick and Nicholas Loman in 2015, they attempted to eradicate and stamp out the West Africa epidemic in Guinea and succeeded in the sequencing of Ebola viruses two days after sample collection [34,35]. Furthermore, Nanopore sequencing has already been applied for the rapid identification of microorganisms [36] and could be used for the detection of antibiotic-resistant pathogens such as *Salmonella* [37].

However, there are still some limitations to long-read technologies. They produce a higher rate of sequencing errors (5–20%) compared to other NGS data (<1%) [38], which are mostly randomly distributed. Nevertheless, long-read technologies are continuously improving, and the error rate is steadily decreasing with new machines. Moreover, bioinformatics algorithms have also evolved and now allow us to generate satisfactory read correction when the sequencing depth is high enough, reaching in some cases an accuracy over 99.9%. Aware of these limitations, the Oxford Nanopore company has refined resolution and throughput sequencing. For this purpose, several Oxford Nanopore products have been developed, including the GridION X5 (Oxford Nanopore Technologies, Oxford, UK) commercialized since March 2017 that can generate up to 100 GB of data per cycle. The PromethION (Oxford Nanopore Technologies, Oxford, UK), a high-throughput desktop device, contains channels for 144,000 nanopores (compared to 512 for the MinIon (Oxford Nanopore Technologies, Oxford, UK). Other platforms are in development, such as the SmidgION (Oxford Nanopore Technologies, Oxford, UK), a sequencer that can be connected to a smartphone and aims to make outdoor sequencing even more accessible.

## 2. Disruption of Clinical Studies on Prokaryotes

The democratization of high-throughput sequencing has made these techniques accessible to many clinical microbiology and public health laboratories. Due to the cost decrease, these structures are equipped with genomics and sequencing platforms or collaborate with external providers. These new resources have changed the way by which hospitals or public health laboratories determine the agents involved in infectious diseases, in addition to the epidemiology and evolution of various infectious pathogens. The following sections describe the main clinical applications of NGS in clinical microbiology and their evolution.

### 2.1. Molecular Detection and Identification of Pathogens

Molecular markers or signatures are small nucleic acid fragments that are specific motifs to the genome of an organism. These signatures make it possible to determine the taxon to which the organism belongs, to predict a restriction profile, to find specific PCR primers or hybridization probes and to develop DNA arrays. The full sequencing of genomes has made it possible to move from a small choice of target sequences such as ribosomal subunits 16S, 23S or housekeeping genes (i.e., rpoB) to a wider choice of sequences, more specific to each biological question. For example, C.R Laing et al. analyzed the 4939 genome sequences of *Salmonella enterica* and identified 404 new subsp. markers in *S. enterica* subsp. [39]. They also identified 1610 universal markers along 10 serovars of *S. enterica* (Typhi, Typhimurium, Enteritidis, Heidelberg, Paratyphi, Kentucky, Agona, Weltevreden, Bareilly and Newport). These new signatures are intended to refine and improve the identification and diagnosis of *S. enterica* strains.

In recent years, the determination of new molecular markers has been facilitated by the massive use of WGS. This provided epidemiologists with a great tool to understand and predict the spread of bacterial species or to study the diversity of bacterial clones and their relationships. A wide genomic study of samples from various locations of a hospital revealed a reservoir of bacterial plasmids conferring carbapenem resistance [40]. The study is part of a large bacterial sequencing project at the Sanger Institute that widely use SMRT Pacific Biosciences (Pacific Biosciences, Menlo Park, CA, USA) technology, leading to sequencing and assembly of over 3000 complete bacterial genomes (from PHE’s National Collection of Type Cultures (NCTC) https://www.phe-culturecollections.org.uk/collections/nctc-3000-project.aspx, accessed on 8 December 2021).

### 2.2. SNPs Genotyping

Genotyping is another strategy for molecular identification. Genotyping is the discipline that aims to determine the identity of a genetic variation for a given organism, at some specific positions, on the whole or only a part of its genome. Current methods of genotyping include restriction fragment length polymorphism identification (RFLPI) of genomic DNA, random amplified polymorphic detection (RAPD) of genomic DNA, amplified fragment length polymorphism detection (AFLPD), polymerase chain reaction (PCR), allele-specific oligonucleotide (ASO) probes, hybridization to DNA microarrays and more recently, DNA sequencing using NGS. The availability of complete genomes due to NGS has made new genotyping methods such as Microsatellites SSR (simple sequence repeats), SNP (Single Nucleotide Polymorphisms) or ISBP (Insertion Site-Based Polymorphisms) possible.

Genotyping by microsatellites SSR is now commonly used to classify isolates from one another. It consists of using tandem repeats in the genomes, called VNTRs (variable number tandem repeats). These repeats are amplified, and the different sizes of the fragments obtained make it possible to determine to which strains they belong.

Genotyping by SNP is also largely used and consists of looking at point mutations for (i) a set of given genes locations (e.g., MultiLocus Sequencing Typing, MLST) or (ii) at the level of the whole genome scale.

(i) MLST allows the characterization of a genus (or species) that is already known to identify the species (or subspecies) thanks to the SNPs comparisons within a set of housekeeping genes [41,42,43]. It is commonly the reference technique to discriminate between different strains. The sequences of these housekeeping genes have the particularity to present a stable polymorphism in time but are divergent enough to distinguish strains between them. MLST analyses have become common because they provide good resolution while being easily reproducible and standardizable. Challagundla et al. analyzed 598 genome sequences of *Staphylococcus aureus* to track the evolution of Clonal Complex 5 Methicillin-Resistant, which caused several hospital-associated infections in the Western hemisphere [44]. Their analysis based on MLST comparisons was able to identify and characterize the geographical spread of S. aureus. CC5-MRSA clones over the world.
(ii) The study of SNPs at the level of complete genomes is obviously more efficient and accurate than using only a set of housekeeping genes. This global approach is being developed at the same time as the WGS is being facilitated. An example is the tracking of diffusion and monitoring the evolution of *M. tuberculosis* Beijing lineage [41], a very virulent and potentially antibiotic-resistant strain. Using a large dataset of a single *M. tuberculosis* lineage, Merker et al. identified the biogeographic structure and evolutionary history of the Beijing lineage worldwide through the SNPs analysis of 4987 isolates from 99 countries [45]. They showed that this lineage originated in the Far East, from where it spread throughout the world in several waves. In addition, global SNPs genotyping was applied to *Mycobacterium abscessus*, a human skin bacterium. Choo et al. described the migration of the clinical isolates through different geographical locations, from India to Southeast Asia, Europe and then to the USA [46]. The outbreak of *Vibrio cholerae* in Haiti [47] is another example of the ability of SNPs genotyping to track strains. Talkington et al. have sequenced 122 isolates, genotyped and compared with isolates from other countries. The authors used SNP analyses to establish phylogeny and trace the origins of these outbreaks. Characterization based on genomes proves that Haiti isolates are clonally and genetically similar to isolates originating in southern Asia and Africa.

### 2.3. Phenotype Prediction to Track Virulence Factors and Antimicrobial Resistance

The current availability of a large number of genomes enables us to achieve a “genome wide association study” (GWAS). GWAS aims to identify significant associations between genetic traits and phenotypes. Regarding microbes, these GWAS studies generally focus on associations between nucleotide polymorphisms (SNPs) and phenotypes. Genome-based phenotypic prediction can relate to the detection of virulence factors. We then speak about “pathogenomics”. Understanding the genetic variations and mechanisms of infectious disease emergence and adaptation holds promise to improve disease prevention, intervention and to develop more targeted therapies [48].

The presence of a virulence factor does not necessarily imply that the bacterium will be pathogenic, and some bacteria may have one or more virulence genes in their genome without providing a pathogenic phenotype. This is illustrated by the study carried out by Armougom et al., which shows that the bacteria *Citrobacter Koseri*, despite possessing the *Pla* gene identical to that of *Yersinia pestis*, does not provide any particular pathogenicity [49]. The prediction of pathogenicity must take into account the whole genome, integrating the possible associations between virulence factors, the presence of other genes that may repress the virulence factors or the structure of the genome itself [50]. Phenotypic prediction can also be used to detect antimicrobial resistance (AMR). Therefore, predicting these resistances from the genomes can be an efficient tool to anticipate and propose treatments. Thus, the complete sequencing of genomes offers the possibility of accurately predicting the potential resistance of various strains [51].

Infections caused by bacteria with AMR are considered a priority by several global public health organizations around the world because they are responsible for high morbidity, mortality and health costs yearly (https://www.cdc.gov/drugresistance/national-estimates.html, accessed on 25 November 2021). The Organisation for Economic Co-operation and Development has estimated that infections with AMR could be responsible for 2.4 million deaths in Europe, North America and Australia in the next 30 years and would cost US$3.5 billion per year (https://read.oecd-ilibrary.org/social-issues-migration-health/stemming-the-superbug-tide_9789264307599-en, accessed on 8 December 2021). Inappropriate prescription of antibiotics is most often responsible for the spread of AMR bacteria, especially in uncomplicated viral infections or with broad-spectrum antibiotics for susceptible bacterial infection. In clinical laboratories, antibiotic susceptibility testing (AST) requires that the bacteria be cultivated, and results are usually obtained by a standard method within 36 h after the patient has been sampled. Recent advances in DNA sequencing technologies have revolutionized microbiology diagnosis, and microbial surveillance, in addition to the routine use of WGS, has become an important tool for surveillance and infection control. In contrast, these technologies have not yet found their place in routine diagnostic microbiology laboratories to characterize AMR in real-time and culture remains the primary method used in clinical laboratories. However, the use of WGS can be a powerful alternative and provide more information. This reveals potential factors for the spread of AMR bacteria in a hospital or in the community and therefore plays a major role in the diagnosis and the treatment of infectious diseases. Citing the example of the colistin *mcr-1* resistance emergence surveillance survey conducted by Falgenhauer and colleagues in 2016 [52], the authors built a database of 577 *Enterobacteriaceae* genomes obtained from different sources (human, animal and environmental), which was queried to identify four previously undiagnosed colistin-resistant isolates. In addition, they demonstrated the existence of multiple horizontal pathways of this resistance. In 2017, Jeukens et al. analyzed 59 sequence genomes of *Achromobacter* genera and identified genes involved in efflux-mediated antibiotic resistance compared with the Comprehensive Antibiotic Resistance Database (CARD) [53]. The resistome analysis showed that the clinical specimens carried more antibiotic resistance genes than other isolates [54].

Virulence factors or AMR genes can be present on chromosome or mobile elements such as plasmids, bacteriophages or transposons, facilitating their spread [55]. The determination of their exact location is thus useful when evaluating the potentiality of the transmission. However, several studies have shown the limits of short-read sequencing for plasmid reconstruction due to the presence of repeats that are sometimes shared with the chromosomal DNA [56]. Today, repetitive regions can be spanned by the use of long-read sequencing technologies [36]. Reliable reconstruction of genome structure is therefore important for generating accurate phenotype predictions. Nguyen M et al. used the whole genome sequences of 1668 clinical isolates of *Klebsiella pneumoniae* and showed that machine learning can be used to construct a reliable, complete Minimal Inhibitory Concentration (MIC prediction) panel for isolates without any previous information about the underlying genetic content or resistance phenotypes of the strains. These studies also show that these phenotype prediction strategies are only effective when the genomes are reconstructed with high quality [57,58,59].

New sequencing technologies are becoming essential to accurately characterize and predict bacterial phenotypes of clinical interest. Their applications offer new tools for diagnosis and prevention at the patient level, but also on a larger scale, such as the prevention of epidemics by identifying virulence factors or resistance genes at an early stage in order to establish the most appropriate strategy.

The characterization of the whole genome is important to establish global phenotype predictions. Long-read technology is, therefore, an essential tool. However, short reads still have the advantage over long reads for phenotypic predictions. Indeed, for predictions that require high-precision SNPs, the sequencing error rate penalizes long reads. Furthermore, some studies have shown that combining the two technologies was possible with many advantages. The Illumina reads are used to correct the long reads, which will then be used for accurate assemblies [55,60,61,62]. This strategy is effective but has the disadvantage of a high cost for routine detection. However, technologies are evolving. As an example, Zhou et al. demonstrate in a recent study, the potential of nanopore sequencing to provide pathogen identification as well as antimicrobial resistance and virulence genes prediction from metagenomic samples [63].

### 2.4. Comparative Genomics to Understand bacterial Strains Evolution

The discovery of genetic variants underlying bacterial phenotypes and the prediction of phenotypic traits are fundamental tasks of bacterial genomics [64,65,66,67,68]. Thus, comparative genomics can be used for the prediction of specific microbial phenotypes for various clinical applications such as characterization of outbreaks, performing phylogeography allowing tracing and monitoring pathogen evolution or analysis of genomic diversity of strains. Comparative genomics corresponds to the comparison of biological information derived from whole-genome sequences and genome reconstructions. Comparative genomics therefore began in 1995, when the first two whole organism genomes, *Haemophilus influenzae* and *Mycoplasma genitalium*, were published [4,69]. Bioinformatics tools then appear that provide a way to compare the genome sequences themselves, RNAs, proteins, and gene annotations that can be derived from them. These tools are constantly evolving to deal with the exponential proliferation of sequenced genomes driven by advances in sequencing technology and to become more comprehensive and user-friendly. The use of comparative genomic approaches is reaching maturity. However, the use of short reads can limit the comparative genomics analysis for microbes. Genomes are rarely fully completed, and even if they are, some assembly uncertainties often remain, which leads to doubts about the final genome structure. This is particularly the case for large genomes, which often contain repeated regions (e.g., operons or repetitive mobile elements) that are difficult to assemble [70]. Furthermore, even if genomes are released as completed on public databases, the comparison of synteny rearrangements between closed species or comparisons of redundant regions are still problematic. Indeed, structural variations (SV) within the genomes play an important role and have to be assembled correctly. SV refers to chromosomal rearrangements typically classified as insertions, inversions, duplications, deletions and translocations describing resulting combinations of DNA losses or gains.

Short-read sequencing is widely used for the identification of single nucleotide variants (SNVs) and small indels [38,71]. However, it could fail to detect larger structural variations properly, especially when several copies of these fragments exist in a genome [38,71]. In addition, the bad positioning of large genomic rearrangements can lead to misinterpreting the structural variants that may occur between the genomes of closed strains [71,72,73,74].

Some genome rearrangements can have a high impact on prokaryotic genomes [75,76], and they are an important source of diversity between relevant strains for human health [77,78]. However, until recently, they were poorly studied because of the limitations of short-read techniques. Long-read sequencing now breaks these limitations and opens the way to the reliable detection of SV. For instance, a recent multiplatform approach carried out by Chaisson et al. showed that the use of long-read sequencing provided a seven-fold increase in SVs detection compared to standard NGS methods [79]. Due to longer fragment lengths, from several kilobases to ultra-long fragments, long-read sequencing technologies are able to cover structural variations (SV) breakpoints or decipher multiple copies with a high level of confidence. This allows the improvment of some clinical diagnoses that were previously unresolved [72,73].

Finally, long-read sequencing technology offers a real efficient alternative to improve the reliability of genome reconstruction. There is, however, another option that includes the use of a ‘hybrid assembly’ approach. Hybrid assembly combines the long reads for structure reconstruction, and shorts reads that provide a low level of sequencing errors [80]. At this time, this approach is the best alternative to construct high-quality complete genomes with a coverage accuracy that resolves the majority of complex genomic structures [17,81].

High-quality genomes make it possible to better understand the punctual genetic variations between bacterial strains or the large genomic rearrangements that can be the cause of complex phenotypic traits, including their population prevalence and their evolutionary origins [82,83,84,85]. Citing the example of the 1002 yeast genomes project (http://1002genomes.u-strasbg.fr/, accessed on 8 December 2021) led by Jackson et al., which successfully characterized the pan-genome of more than 1000 *S. cerevisiae* isolates worldwide thanks to a hybrid sequencing approach (Pacbio + HiSeq) [86]. This huge set of genomes enabled the discovery of large-scale structural variants that completely refine the phylogenetic relationships and co-evolution along these strains.

A hybrid approach can also be developed using Illumina (San Diego, CA, USA) in association with ONT Nanopore. Even if the Nanopore produce more errors than PacBio (Pacific Biosciences, Menlo Park, CA, USA), new bioinformatics tools help to correct these errors using the Illumina (San Diego, CA, USA) short-reads [87,88,89,90,91]. For instance, Ben Khedher et al. succeed in assembling and characterizing a collection of *Bacillus cereus* group strains by sequencing strains using ONT (Oxford Nanopore Technologies, Oxford, UK) and Illumina HiSeq X Ten (San Diego, CA, USA) [92]. The genomes were improved and refined with a strategy involving a collection of bioinformatics tools [93,94,95] that produce complete and circular chromosomes and plasmids.

### 2.5. Taxogenomics

The large number of complete microbial genomes obtained with NGS has profoundly revolutionized taxonomic analyses. Phenotypic traits have been replaced by nucleotide sequences for taxonomic determination. Initially, based on housekeeping genes such as the rRNA 16S, modern taxonomy is now increasingly based on the whole genome rather than on a few selected genes. Indeed, the classification of taxa based on a single gene such as the 16S may not be discriminatory enough to distinguish closed species such as those of the genus *Aeromonas*, *Pseudomonas*, *Streptococcus* [96]. Therefore, a set of seven universal genes present in all species of the study group was recommended [97] for phylogenetic studies using multilocus sequence analysis (MLSA) or a modification of the multilocus sequence typing procedure (MLST) [98].

At the same time, experimental analyses, such as DNA–DNA hybridizations that were used to differentiate species, were replaced by in silico hybridizations; citing DNA–DNA numerical hybridizations (HDDD) as reference standards for genomic-to-genomic distance (GGDC) or nucleotide averages (ANI) [99,100,101]. More recently, Parks et al. proposed a new standardized bacterial taxonomic approach (GTDB taxonomy) based on genome phylogeny with the analysis of amino acid sequences of 120 proteins encoded by 120 universal genes [102]. They included genomes assembled from metagenomes (MAGs) to increase the diversity of bacterial species cultivated so far. Taking into account a larger part of the genomes content, or the total, substantially contribute to modern bacterial taxonomy and is now known as the “taxogenomics” approach.

This new approach has contributed to differentiating many species and thus participates in discovering many new taxa [103]. For example, Patil PP et al. highlighted the importance of genome-based taxonomy approaches to delineate bacterial species [104]. They have identified cryptic genome species, which are associated with the clinical isolates of *S. maltophilia* and are potentially novel species associated with human infections. Taxogenomics is complementary to other techniques such as phenotypic characteristics descriptions and the proteomic information obtained by MALDI-TOF MS. This approach contributes to the improvement of clinical diagnosis and for the understanding of some specific behavior of infections due to poorly known bacterial species.

Phylogenomics refers to the application of genomics as a means of taxonomic analysis. Phylogenetic tree reconstruction is based on GWAS and aims to improve or refine the taxonomic relatedness between different species. Therefore, phylogenomics facilitates the correct assignment or reassignment of several bacterial genera and species. Thus, several studies have revealed inconsistencies in species classification using a phylogenomic approach. For example, Saati-Santamaría et al. applied a phylogenomic approach to revise the taxonomic organization of the genus *Pseudomonas* and other genera of the Pseudomonadaceae family [105]. The authors proposed the reclassification of some *Pseudomonas* species into the genera *Chryseomonas*, *Stenotrophomonas* and *Xanthomonas* and the creation of three novel genera to encompass several species included in the genus Pseudomonas. Taxogenomics is also a powerful tool for distinguishing clades and thus evolutionary relationships. Gupta et al. conducted comparative and comprehensive phylogenomic analyses on the genome sequences of *Bacillus* species to robustly delineate the different homogeneous clades in phylogenetic and molecular terms [106]. They analyzed genome sequences to identify novel molecular characteristics in the form of conserved signature indels (CSIs) shared by the members of *Bacillus* species clades. As a result, they reported 31 unique CSIs shared by the members of the Subtilis clade or the Cereus clade. Additionally, Radhey S. Gupta et al. proposed 17 *Bacillus* species clades that should be recognized as novel genera based on the phylogenetic and molecular evidence.

### 2.6. Metagenomics

The evolution of NGS has allowed a drastic deployment of the metagenomics field, in particular for the human microbiotas such as intestinal microorganism populations. Along with this microbiota, microorganisms form very diverse communities, and a characteristic of these communities is that a few taxa dominate them, while a very large number of species co-occur with lower frequency. Furthermore, species that cannot be cultivated may also occur and therefore cannot be addressed by classical methods. Knowing that more than 99% of prokaryotes in the environment cannot be cultured in the laboratory, the Metagenomics approach is the culture-independent analysis of a mixture of microbial genomes based on sequencing [107,108]. Even when a culture of microorganisms is possible, metagenomics offers a significant advantage as it allows results to be obtained in only a few hours, whereas it can take several days to obtain results using culture methods.

The rapidly growing interest in microbiome research has been reinforced by the ability to profile different microbial communities using NGS. This culture-free, high-throughput technology enables the identification and comparison of entire microbial communities. Metagenomics typically involves two different sequencing strategies: the first sequencing strategy is termed amplicon metagenomics, which usually utilizes regions as a phylogenetic marker such as the 16S rRNA gene for bacterial communities or the Internal Transcribed Spacer (ITS) region for fungal communities, while the second sequencing strategy is termed shotgun metagenome, and is a whole-genome sequencing approach s (i.e., metagenome-assembled genomes [MAGs]). Samples with high microbial diversity and limited sequencing depths result in observable MAGs representing only a fraction of the shotgun metagenomes actually present. However, MAGs have the advantage over amplicon-based metagenomics to eliminate possible biases due to the amplification of a single genomic region.

Hilton et al. compared two sets of sequencing data, one from metagenomics by amplicons (16 rRNA) and one from whole metagenomic shotgun (WMGS), in their respective abilities to match the same diagnosis as the traditional culture method for patients with ventilator-associated pneumonia (VAP) [109]. The metagenomic analysis was able to produce the same diagnosis as culture methods at the species-level for five of the six samples, while the metataxonomic analysis was only able to produce results with the same species-level identification as a culture for two of the six samples. These results indicate that metagenomic analyses have the accuracy needed for a clinical diagnostic tool, but full integration in diagnostic protocols is contingent on technological improvements to decrease turnaround time and lower costs. Currently, the application of metagenomics in clinical research includes a variety of syndromes of infectious disease diagnostics [110,111,112,113,114,115]. Metagenomics is usually used as a potential tool of microbiome characterization under the analysis of bacterial diversity. For example, Langelier et al. performed a metagenomic analysis on tracheal aspirates from 92 adults with acute respiratory failure. They assessed the airway microbiome, pathogens, and host transcriptome [116]. Through their metagenomics analysis, they provided evidence to determine whether pneumonia illness is infectious or non-infectious. They showed that patients with culture-proven infection had significantly less diversity in their respiratory microbiome.

In recent years, sequencers have considerably increased sequencing depth (i.e., Illumina 10X (San Diego, CA, USA)) leading to the retrieval of rare and underrepresented microbial populations, which were previously difficult or impossible to detect. More recently, long-read sequencers make it possible to consider the partial or complete assemblies of genomes from a whole-genome sequencing approach.

This has led to the discovery of uncultivable bacteria from various microbiota samples, such as the species *Akkermansia muciniphila* [117]. Metagenomics is still in constant development thanks to the contribution of long-read technologies. For example, Somerville et al. tested the feasibility of a complete de novo metagenome-assembled genome (MAGs) from low-complexity microbiomes in a natural microbial community (of natural whey starter cultures (NWCs) used in cheese production) using long-read single-molecule sequencing data [118]. Two NWCs from Swiss Gruyère producers were subjected to whole metagenome shotgun sequencing using a combination of Illumina Miseq (San Diego, CA, USA), PacBio (Pacific Biosciences, Menlo Park, CA, USA) and Oxford Nanopore Technologies MinION (Nanopore, Oxford, UK) to resolve repeat regions. They succeeded to achieve the complete assembly of all dominant bacterial chromosomes, bacterial plasmids and phages and a corresponding prophage. With the help of long-read sequencing, Somerville and his colleagues successfully covered both intra-genomic and inter-genomic repeats, which enabled them to discover biologically relevant information by linking plasmids and phages to their respective host genomes. These findings were obtained by detecting DNA methylation motifs on plasmids without the pre-treatment of the DNA (e.g., bisulfite conversion) and matching prokaryotic CRISPR spacers and their proto-spacers on phages. They illustrated that PacBio (Pacific Biosciences, Menlo Park, CA, USA) and ONT sequencing technologies were crucial instrumentals to achieve MAGs with the possibility to associate plasmids with their most likely bacterial host which was impossible to achieve using only short-reads. So far, WMGS studies have mainly relied on long-read sequencing, establishing that read length is essential for assigning the correct taxon and providing insight into different taxonomic groups during metagenomic analyses [119,120].

### 2.7. Transcriptomics and MetaTranscriptomics

Transcriptomics is the technique used to study an organism’s transcriptome, the sum of all of its RNA transcripts. Unlike the genome, the transcriptome is dynamic and actively evolving. Indeed, the transcriptome produced by a cell is dependent on its activity at a given time. The transcriptome makes it possible to identify genes that are differentially expressed in distinct cell populations or in response to different treatments. Determination of transcripts present in a sample is currently mainly performed by RNA-Seq methods. RNAs extracted from a given organism are converted into cDNAs which are then sequenced, identified and quantified by aligning them to a reference genome or a reference transcriptome. RNA-Seq techniques have seen broad application across diverse areas of biomedical research, including gene expression quantification changes, the prediction of antibiotic resistance, revealing the host–pathogen immunity interactions and the identification of novel virulence factors [115,121,122,123,124,125]. Transcriptomic analysis is also of interest for improving infection control measures and targeted, individualized treatment [126,127].

Hao Van et al. carried out global comparative transcriptomic and genomics analysis between *Campylobacter hepaticus* recovered from the bile of Spotty Liver Disease (SLD) infected chickens and *C. hepaticus* grown in vitro [128]. The transcriptomic analysis revealed how the bacteria adapt to proliferate in the challenging host environment. Additional biochemical experiments confirmed some in silico metabolic predictions. The analysis also indicated that gene clusters associated with glucose utilization, hydrogen metabolism and sialic acid modification as a stress response may play an important role in the pathogenicity of *C. hepaticus*. In addition, directed by transcriptomics and genomics comparison, Hao Van et al. have identified the in vivo transcriptome pattern of *C. hepaticus,* which harbors a wide range of potential virulence factors.

Metatranscriptomics, on the other hand, takes into account all the transcripts of a cell’s population, which may be composed of several thousand different species. The metatranscriptomic can be used to survey the gene functions and regulations of a microbial community at the population scale. This enables the deciphering of microbe–microbe and host–microbe interactions and their responses to environmental stresses. This approach can reveal specific expression profiles even from complex microbial communities. It also has a promising future for the discovery of new proteins such as biocatalysts of pharmaceutical interest.

Metatranscriptomic sequencing provides direct access to culturable and non-culturable microbial transcriptome information by large-scale, high-throughput sequencing of transcripts from all microbial communities in specific environmental samples. Metatranscriptomic sequencing offers an opportunity to randomly sequence mRNAs as a unit for understanding the regulation of complex processes in microbial communities. The study of the metatranscriptome through next-generation sequencing techniques allows us to obtain gene expression profiles from whole microscopical populations, providing new insights into poorly known biological systems and overcoming technical limitations related to individual bacteria isolation. Long-read technologies have improved transcriptomics analysis by allowing the sequence of full-length transcripts and thus avoiding assemblies that may give errors. The complexity of metatranscriptomes is particularly challenging, and long reads have greatly assisted in deciphering the high sequence similarity of highly abundant RNA species such as rRNAs or possible alternatively spliced isoforms and their distinct expression levels. In addition, long-read technology, especially MinION technology, has illustrated its power in the accurate quantification of transcripts, allele-specific expression and single cell expression profiling, examining clonal heterogeneity in gene expression and thus potentially revolutionizing our understanding of the repertoire and functions of immunological cell receptors [129,130,131,132]. The use of short reads still has the advantage of sequencing depth, but this gap is narrowing. Moreover, short reads can also be combined with long reads to further improve performance in transcriptome analysis [133].

## 3. Discussion

Since the first high-throughput sequencing machines in the early 2000s, the machines have never stopped evolving. For each new generation, companies offer a better sequencing depth or longer read lengths at lower costs. Bacteriology, whether for fundamental or clinical purposes, has greatly benefited from these technological advances, with genomics as a central approach.

The huge amount of data generated remains a challenge, and the support of powerful bioinformatics tools is necessary to face it. New bioinformatics tools make the evaluation of the results produced less complex by presenting summaries that are accessible to non-specialists. Similarly, new computing technologies related to storage or calculation can help to manipulate the data. Recent cloud systems now allow storage or long computations on remote servers without the need to invest in expensive local solutions.

Long-read sequencers have appeared more recently. These technologies have solved bottlenecks due to short fragments that limited, for example, the reliable and complete reconstruction of genomes. Complex and repetitive regions of the genome can be partially or in totality solved. De novo genome assemblies have thus been facilitated. Genomes sequenced with long reads better reflect reality, resulting in the greatly improved reliability of genomic studies aimed at the evolution of pathogens, drug resistance or genetic diversity such as that due to structural variants.

These long-read technologies are recent and still under development. They were, until recently, relatively expensive compared to short reads, but the arrival of ONT Nanopore (Oxford Nanopore Technologies, Oxford, UK) has shown that it is possible to use long-reads for reasonable costs while requiring minimal sample preparation before sequencing. This democratization makes routine clinical applications such as diagnostics or personalized medicine possible.

ONT continues its developments, closely followed by competitors such as PacBio (Pacific Biosciences, Menlo Park, CA, USA), which now offer more affordable devices. Recently, ONT has opened the way to peptide sequencing methods using the same principle [134,135]. ONT’s developments are also focused on reducing or even eliminating the use of chemical reagents to prepare the libraries. Thus, the expertise required before sequencing is reduced to a minimum. For example, it is possible via ONT to sequence a sample directly without a DNA extraction procedure, and this can even be conducted outside a laboratory directly in the field. Data can be transmitted in real-time for analysis by connecting the device to a small computer or even directly via the internet to be analyzed on a dedicated cloud platform.

Real-time nucleotides sequencing analysis of the DNA strand is a promising method because it has many advantages. Indeed, it makes the “Read until” method or “selective sequencing in real-time” possible. The principle of nanopore sequencing is used to simultaneously pass DNA strands through small pores arranged on a membrane. The real-time analysis allows knowing the nucleotides sequence even before the strand has finished its passage in the pore. The software can then detect the beginning of the sequence, and if this sequence does not correspond to the target sequences expected by the user, the passage in the pore can be interrupted to leave the place to another molecule of the sample. In the end, the sequencing is largely optimized since all the sequences without any interest will not have been taken into account, leaving room for better coverage of the other relevant or user-defined regions. The post analysis is also facilitated since there will be only a few undesirable sequences. Selective sequencing in real-time also has advantages for de novo assembly because it allows us to not over-sequence certain regions. This has the effect of homogenizing the coverage on the whole genome and thus provides a correct depth for almost all regions.

The combination of long-reads and selective sequencing also means that fewer computer resources are needed to process the files because the data produced is less redundant. On the other hand, real-time analysis requires new resources to evaluate all strands at the same time. This parallelization requires important resources but has been partially solved thanks to the use of GPUs (graphical processor units). GPUs are the processors in video cards that have become extremely powerful in recent years to satisfy increasingly detailed and immersive video games. The interest of GPUs is that they can perform highly parallelized tasks in a very fast way. Exploiting these capabilities has been a boon for real-time analysis of sequences, especially since these video cards, are quite modest in cost (usually less than €1500) compared to an equivalent dedicated computing cluster.

The implication of GPUs in the long-read sequencing landscape has also been of great help with the concomitant arrival of new parallelized long-read sequencers. Indeed, other versions of the Nanopore platform have been introduced, such as the GridION (Oxford Nanopore Technologies, Oxford, UK), the PromethION (Oxford Nanopore Technologies, Oxford, UK) and the new MinION Mk1C platform (Oxford Nanopore Technologies, Oxford, UK), which offer higher throughput thanks to the use of sequencing cells arranged in parallel. In addition, Nanopore proposes solutions that combine a sequencer and a computer with a GPU. This is the case with their new platform, the MinION Mk1C. This device combines real-time sequencing of long-reads, high throughput and connectivity to a powerful computer with GPU. This new platform is proposed as an all-in-one portable device that can be used in any environment with a 4G internet connection. It does not require any accessories to generate and analyze the data produced.

For its part, PacBio (Pacific Biosciences, Menlo Park, CA, USA) has also been evolving its machines, including the “Sequel IIe” system, which reduces data processing, has higher throughput than previous machines and an even lower error rate. To achieve this, in 2019, PacBio (Pacific Biosciences, Menlo Park, CA, USA) redesigned a new circular consensus sequencing (CCS) model [136]. With this new technology, individual DNA strands are converted into closed loops that can be repeatedly read. These repeated reads eliminate random errors and provide highly accurate results. Circular consensus sequencing (CCS) has evolved from single-molecule real-time sequencing (SMRT) technology to another type of long read, known as a highly accurate read, or “HiFi Read”. These data produce consensus reads over 25 kb and provide base-level resolution with >99.9% single molecule read accuracy. Consequently, it can produce reference-quality de novo assemblies by generating complete, contiguous and correct assemblies of various genome types, even for large and complex bacterial genomes. HiFi reads allow precise detecting of all the structural and other types of variations that cannot be identified with other technologies. Characterization and annotation of the entire transcriptome are now also possible with HiFi reads to identify complex alternative splicing events.

The amount of digital data produced by NGS is such that powerful computer infrastructures are required, both in terms of storage and computing power. However, it can be difficult for non-specialists to deal with. To overcome this, several tools and platforms have been developed to improve data management by automating some analyses and by offering graphical interfaces that facilitate access to files and results [137,138,139,140,141,142,143,144]. These data managers often integrate a wide variety of tools and workflows to accelerate the most-used operations on NGS data, such as quality control, alignment and variant calling.

Among these platforms, the best known is certainly Galaxy [137,138]. This system provides a web interface to many common bioinformatics programs, allowing users to perform files manipulation and analysis without the need to go through command lines.

Other systems such as Omnics Pipe [139] are more for users who need to analyze a large dataset and automate data analysis pipelines for multiple NGS technologies. It includes a set of bioinformatics tools that can be combined in predefined pipelines. HTS-FLOW is another popular management system developed by the Instituto Italiano di Tecnologia in Milano [140]. HTS-FLOW manages NGS analysis in a traceable way through a graphical interface, produces data in standard locations associated with metadata and script analyses. A recent tool, the One Touch Pipeline (OTP) [141], is a platform for structured data storage and NGS data management developed by the German Cancer Research Center (DKFZ). It is designed to graphically manage routine NGS analyses in a scalable and automated manner, from importing raw sequence data to notifying project members of the completion of their analyses to aligning and identifying genomic events.

Standardization of analyses is probably the main challenge of omics studies. New technologies and new bioinformatics tools appear regularly and in an unprecedented way, making it difficult to converge into similar analysis methods. The choice of bioinformatics methods and algorithms to be implemented implies finding a balance between computational speed, sensitivity and time allowed for the analysis. Most bioinformatics tools are open-source and freely available through platforms such as GitHub, GitLab or SourceForge. Some authors have tried to establish catalogs of tools commonly used in a particular field, such as for the use of long-read sequencing data (https://github.com/B-UMMI/long-read-catalog, accessed on 8 December 2021). However, it remains difficult to find the best strategy, and the experience of the bioinformatician will often be essential to choose the appropriate tools, parameters and their correct applications.

## 4. Conclusions

Today, it seems obvious that, whatever technology is imposed on the market, the future of sequencing will be turned towards long reads or even reads that can represent the entirety of a chromosome or a mobile element. In this case, it will no longer be necessary to facilitate assembly. Costs will also obviously continue to fall, making these new technologies more and more common. Sample preparation is simplified with each new generation, and already manufacturers such as Nanopore propose to simply place a sample on the sequencer chip. In addition, the automatisation of analysis methods is also developing rapidly. The biologist or clinician can quickly obtain an overview of the results in an intelligible way without needing bioinformatics skills. More advanced analyses requiring bioinformatics skills will still be necessary in some cases, especially for more fundamental projects or those requiring more investigation. However, routine clinical applications can often be satisfied with the results produced through in-line platforms to which the sequencers are connected. These cloud platforms integrate pipelines that automate data processing by software suites, and the results are graphically displayed and standardized.

Finally, similar to the first computers, sequencers have largely decreased in size and can, for some models, be transported directly to the field. Often associated with large computers such as computing clusters, it is now possible to perform routine analyses and real-time sequencing from a simple laptop computer equipped with a good video card. The quality and quantity of information produced by these machines will continue to increase, leading to a better understanding of the biological mechanisms governing the functioning of microorganisms.

## Figures and Tables

**Table 1:** Summary of the main advantages of long-reads sequencing over short-read sequencing.

Short-Read Technologies	Long-Read Technologies
Fixed run time: - Increased time to results and inability to identify workflow errors before completed sequencing - Additional practical complexities associated with handling and storing large volumes of sequence data	Real-time data acquisition: - Achieve rapid turnaround with immediate access to results - Enrich single targets during sequencing, with no additional sample prep using adaptive sampling - Identify microbiome composition and resistance in real-time
Limited flexibility: - Sample batching often required for optimal efficiency - Potentially leads to long turnaround times - Benchtop devices confine sequencing to centralized locations	Scalable and flexible: - Scale to suit the throughput needs - Decentralize sequencing - No sample batching needed
Read length typically 50–300 bp	Unrestricted read length (>4 Mb achieved)
Limited genomic characterization: - Short reads do not span entire structural variants or important classes of genomic aberrations (repeat expansions and repeat-rich regions) - fragmented genome assemblies and ambiguous isoforms identification - Short sequencing reads may not span complex genomic regions such as genes duplications, transposons and prophage sequences - Potentially missing important genomic information	Comprehensive genomic characterization: - Identify mutations in complex and repetitive genomic regions - Accurately phase single nucleotide variants, structural variants, and base modifications - Can fully assemble genomes more easily - Simplify de novo assembly and correct microbial reference genomes - Possibility to completely assemble genomes and plasmids from metagenomic samples - Resolving complex genomic regions and similar species
Amplification required: - Amplification can introduce bias reducing uniformity of coverage and removes base modifications - Necessitating additional sample prep and sequencing runs	Amplification-free protocols: - Detect and phase base modifications as standard - No additional preparation required
Constrained to the lab: - Traditional sequencing technologies are typically expensive and require substantial site infrastructure - Usually limited its usage to well-resourced environments - Delay in transmitting the results	Sequence anywhere: - Sequence in your lab or in the field - Sequence at sample source and eliminate sample shipping delays - Scale-up with high-throughput
